# 4-Chloro­anilinium 4-methyl­benzene­sulfonate

**DOI:** 10.1107/S160053681104712X

**Published:** 2011-11-12

**Authors:** Jerry P. Jasinski, James A. Golen, A. S. Praveen, H. S. Yathirajan, B. Narayana

**Affiliations:** a Department of Chemistry, Keene State College, 229 Main Street, Keene, NH 03435-2001, USA; b Department of Studies in Chemistry, University of Mysore, Manasagangotri, Mysore 570 006, India; c Department of Studies in Chemistry, Mangalore University, Mangalagangotri, 574 199, India

## Abstract

In the crystal structure of the title salt, C_6_H_7_ClN^+^·C_7_H_7_O_3_S^−^, the cations and anions are linked *via* N—H⋯O hydrogen bonds into double chains in [101]. Weak inter­molecular C—H⋯π-ring inter­actions link these chains into layers parallel to the *ac* plane.

## Related literature

For background literature concerning mol­ecular–ionic compounds, see: Czupinski *et al.* (2002 ▶); Katrusiak & Szafranski (2006 ▶). For related structures, see: Chanawanno *et al.* (2009 ▶); Chantrapromma *et al.* (2010 ▶); Collier *et al.* (2006 ▶); Fun *et al.* (2010 ▶); Kobkeatthawin *et al.* (2009 ▶); Li *et al.* (2005 ▶); Lin, (2010 ▶); Rahmouni *et al.* (2010 ▶); Smith *et al.* (2009 ▶); Tabatabaee & Noozari, (2011 ▶); Wu *et al.* (2009 ▶); Zhang & Liu (2010 ▶).
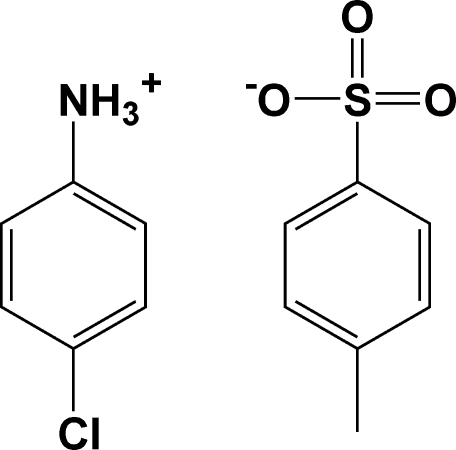


## Experimental

### 

#### Crystal data

C_6_H_7_ClN^+^·C_7_H_7_O_3_S^−^
*M* *_r_* = 299.76
Triclinic, 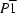

*a* = 5.7253 (5) Å
*b* = 7.5160 (6) Å
*c* = 15.7642 (13) Å
α = 95.166 (6)°
β = 96.148 (7)°
γ = 92.353 (7)°
*V* = 670.83 (10) Å^3^
*Z* = 2
Mo *K*α radiation
μ = 0.44 mm^−1^
*T* = 173 K
0.40 × 0.20 × 0.12 mm

#### Data collection

Oxford Diffraction Xcalibur Eos Gemini diffractometer
Absorption correction: multi-scan (*CrysAlis RED*; Oxford Diffraction, 2010 ▶) *T*_min_ = 0.843, *T*_max_ = 0.949
5280 measured reflections
3439 independent reflections
3144 reflections with *I* > 2σ(*I*)
*R*_int_ = 0.017

#### Refinement

*R*[*F* ^2^ > 2σ(*F* ^2^)] = 0.032
*wR*(*F* ^2^) = 0.088
*S* = 1.08
3439 reflections
182 parameters
6 restraints
H atoms treated by a mixture of independent and constrained refinement
Δρ_max_ = 0.42 e Å^−3^
Δρ_min_ = −0.45 e Å^−3^

### 

Data collection: *CrysAlis PRO* (Oxford Diffraction, 2010 ▶); cell refinement: *CrysAlis PRO*; data reduction: *CrysAlis RED* (Oxford Diffraction, 2010 ▶); program(s) used to solve structure: *SHELXS97* (Sheldrick, 2008 ▶); program(s) used to refine structure: *SHELXL97* (Sheldrick, 2008 ▶); molecular graphics: *SHELXTL* (Sheldrick, 2008 ▶); software used to prepare material for publication: *SHELXTL*.

## Supplementary Material

Crystal structure: contains datablock(s) global, I. DOI: 10.1107/S160053681104712X/bt5699sup1.cif

Structure factors: contains datablock(s) I. DOI: 10.1107/S160053681104712X/bt5699Isup2.hkl

Supplementary material file. DOI: 10.1107/S160053681104712X/bt5699Isup3.cml

Additional supplementary materials:  crystallographic information; 3D view; checkCIF report

## Figures and Tables

**Table 1:** Hydrogen-bond geometry (Å, °) *Cg*2 is the centroid of the C8–C13 ring.

*D*—H⋯*A*	*D*—H	H⋯*A*	*D*⋯*A*	*D*—H⋯*A*
N1—H1*NC*⋯O1^i^	0.92 (1)	2.02 (1)	2.8579 (16)	151 (2)
N1—H1*NC*⋯O1	0.92 (1)	2.42 (2)	3.0814 (16)	129 (1)
N1—H1*NB*⋯O3^ii^	0.92 (1)	1.88 (1)	2.7940 (15)	175 (2)
N1—H1*NA*⋯O2^iii^	0.93 (1)	1.98 (1)	2.8764 (15)	163 (2)
C2—H2*A*⋯*Cg*2^i^	0.95	2.91	3.5340 (16)	124

Symmetry codes: (i) 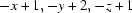; (ii) 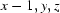; (iii) 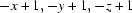.

**Fig. 1.**
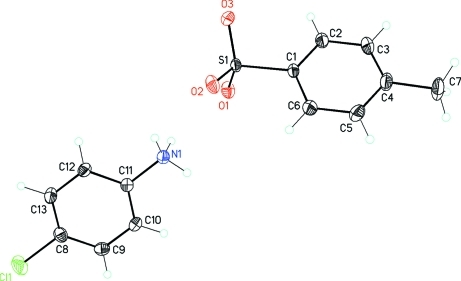
Molecular structure of the title compound showing the atom labeling scheme and 30% probability displacement ellipsoids.

**Fig. 2.**
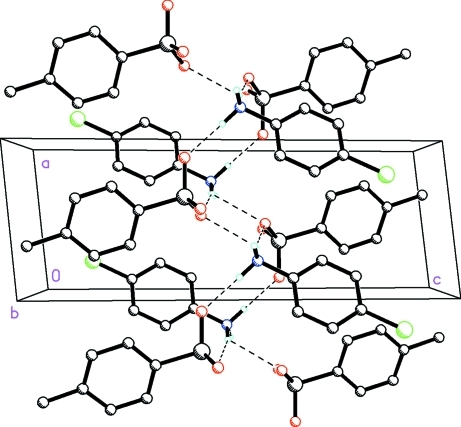
Packing diagram of the title compound viewed along the b axis. Dashed lines indicate N—H···O hydrogen bonds forming infinite 1-D chains along the c axis.

## supplementary crystallographic information

### Comment

A variety of pharmaceutical drugs are prepared as salts of benzenesulfonic acid and are known as besylates. Recently much attention has been devoted to simple molecular–ionic crystals containing organic cations and anions due to the tunability of their special structural features and their interesting physical properties (Czupinski *et al.*, 2002; Katrusiak & Szafranski, 2006). In the title compound, the proton of the sulfonic group of sulfonic acid has been transferred to the N atom of the 4-chloroaniline molecule, leading to the formation of the molecular complex, (I).

Crystal structures of some benzenesulfonate derivatives, viz., 2,4,6-triamino-1,3,5-triazin-1-ium 4-methylbenzenesulfonate monohydrate (Li *et al.*, 2005), ephedrine besylate (Collier *et al.*, 2006), 2-ethyl-6-methylanilinium 4-methylbenzenesulfonate (Wu *et al.*, 2009), 2-[(E)-2-(4-ethoxyphenyl)ethenyl]-1-methylpyridinium 4-methylbenzenesulfonate monohydrate (Chanawanno *et al.*, 2009), (E)-2-[4-(dimethylamino)styryl]-1-methylquinolinium 4-methylbenzenesulfonate monohydrate (Kobkeatthawin *et al.*, 2009), 4-chloroanilinium 2-carboxy-4,5-dichlorobenzoate (Smith *et al.*, 2009), 4-chloroanilinium (4-chlorophenyl)guanidinium dichloride hemihydrates (Zhang & Liu, 2010), 4-chloroanilinium hydrogen oxalate hemihydrates (Rahmouni *et al.*, 2010), 4-(cyanomethyl)anilinium 4-methylbenzene sulfonate monohydrate (Lin, 2010), 1-methyl-2-[(E)-2-(2-thienyl)etheny] quinolinium 4-bromobenzenesulfonate (Fun *et al.*, 2010), (E)-2-[4-(dimethylamino)styryl]-1-methylpyridinium 4-methylbenzenesulfonate monohydrate (Chantrapromma *et al.*, 2010), 2-aminopyrimidin-1-ium 4-methylbenzenesulfonate (Tabatabaee & Noozari, 2011), have been reported.  In view of the importance of benzenesulphonic acid, we report herein the crystal structure of the title compound (I).

In the crystal structure of the title salt, C_6_H_7_ClN^+^. C_7_H_7_O_3_S^-^, (Fig. 1) the cations and anions are linked *via* N—H···O hydrogen bonds into doubled chains in [101] (Fig. 2).  Weak intermolecular C—H···Cg2 π-ring interactions (table 1) link further these chains into layers parallel to the *ac* plane. [Cg2 = C8—C13 centroid]

### Experimental

4-methylbenzenesulfonic acid monohydrate (1g, 5.25 mmol ) was added to a stirred solution of 4- chloroaniline (0.67 g, 5.25 mmol ) in methanol ( 10 mL). The resulting suspension was dissolved in chloroform (10 ml) and stirred at 323 K for 10 minutes and cooled to room temperature to afford the title compound (I). Single crystals were grown from a mixture of chloroform and methanol by the slow evaporation method (m.p.: 524-532 K).

### Refinement

H1NA, H1NB and H1NC were located by a Fourier map and refined isotropically (for N1, dfix = 0.94 (2)Å; dang = 1.50 (2)Å).  All of the remaining H atoms were placed in their calculated positions and then refined using the riding model with Atom—H lengths of 0.95 (CH) or 0.98Å (CH_3_). Isotropic displacement parameters for these atoms were set to 1.18-1.21 (CH) or 1.51 (CH_3_) times *U*_eq_ of the parent atom.

### Crystal data

**Table:**

C_6_H_7_ClN^+^·C_7_H_7_O_3_S^−^	*Z* = 2
*M**_r_* = 299.76	*F*(000) = 312
Triclinic, *P*1	*D*_x_ = 1.484 Mg m^−^^3^
Hall symbol: -P 1	Mo *K*α radiation, λ = 0.71073 Å
*a* = 5.7253 (5) Å	Cell parameters from 2538 reflections
*b* = 7.5160 (6) Å	θ = 3.6–29.9°
*c* = 15.7642 (13) Å	µ = 0.44 mm^−^^1^
α = 95.166 (6)°	*T* = 173 K
β = 96.148 (7)°	Block, colorless
γ = 92.353 (7)°	0.40 × 0.20 × 0.12 mm
*V* = 670.83 (10) Å^3^	

### Data collection

**Table:**

Oxford Diffraction Xcalibur Eos Gemini diffractometer	3439 independent reflections
Radiation source: Enhance (Mo) X-ray Source	3144 reflections with *I* > 2σ(*I*)
graphite	*R*_int_ = 0.017
Detector resolution: 16.1500 pixels mm^-1^	θ_max_ = 30.0°, θ_min_ = 3.6°
ω scans	*h* = −7→7
Absorption correction: multi-scan (*CrysAlis RED*; Oxford Diffraction, 2010)	*k* = −9→9
*T*_min_ = 0.843, *T*_max_ = 0.949	*l* = −22→21
5280 measured reflections	

### Refinement

**Table:**

Refinement on *F*^2^	Primary atom site location: structure-invariant direct methods
Least-squares matrix: full	Secondary atom site location: difference Fourier map
*R*[*F*^2^ > 2σ(*F*^2^)] = 0.032	Hydrogen site location: inferred from neighbouring sites
*wR*(*F*^2^) = 0.088	H atoms treated by a mixture of independent and constrained refinement
*S* = 1.08	*w* = 1/[σ^2^(*F*_o_^2^) + (0.0406*P*)^2^ + 0.2844*P*] where *P* = (*F*_o_^2^ + 2*F*_c_^2^)/3
3439 reflections	(Δ/σ)_max_ = 0.001
182 parameters	Δρ_max_ = 0.42 e Å^−^^3^
6 restraints	Δρ_min_ = −0.45 e Å^−^^3^

### Special details

**Table:**

Geometry. All esds (except the esd in the dihedral angle between two l.s. planes) are estimated using the full covariance matrix. The cell esds are taken into account individually in the estimation of esds in distances, angles and torsion angles; correlations between esds in cell parameters are only used when they are defined by crystal symmetry. An approximate (isotropic) treatment of cell esds is used for estimating esds involving l.s. planes.
Refinement. Refinement of *F*^2^ against ALL reflections. The weighted *R*-factor *wR* and goodness of fit *S* are based on *F*^2^, conventional *R*-factors *R* are based on *F*, with *F* set to zero for negative *F*^2^. The threshold expression of *F*^2^ > σ(*F*^2^) is used only for calculating *R*-factors(gt) *etc*. and is not relevant to the choice of reflections for refinement. *R*-factors based on *F*^2^ are statistically about twice as large as those based on *F*, and *R*- factors based on ALL data will be even larger.

### Fractional atomic coordinates and isotropic or equivalent isotropic displacement parameters (Å^2^)

**Table:**

	*x*	*y*	*z*	*U*_iso_*/*U*_eq_	
S1	0.64768 (6)	0.72752 (4)	0.391167 (19)	0.01562 (9)	
Cl1	−0.20941 (8)	0.85567 (6)	0.86957 (3)	0.03622 (12)	
O1	0.56763 (19)	0.89688 (13)	0.42702 (6)	0.0227 (2)	
O2	0.52819 (19)	0.57587 (13)	0.42253 (6)	0.0231 (2)	
O3	0.90320 (18)	0.71964 (16)	0.40103 (6)	0.0281 (2)	
N1	0.2327 (2)	0.74505 (16)	0.54631 (7)	0.0179 (2)	
H1NC	0.329 (3)	0.8434 (17)	0.5411 (11)	0.022*	
H1NB	0.123 (2)	0.730 (2)	0.4992 (10)	0.022*	
H1NA	0.323 (3)	0.6460 (18)	0.5467 (11)	0.022*	
C1	0.5595 (2)	0.71390 (16)	0.27970 (8)	0.0150 (2)	
C2	0.7106 (2)	0.7789 (2)	0.22507 (9)	0.0219 (3)	
H2A	0.8638	0.8264	0.2470	0.026*	
C3	0.6356 (3)	0.7737 (2)	0.13818 (9)	0.0277 (3)	
H3A	0.7385	0.8182	0.1007	0.033*	
C4	0.4120 (3)	0.7044 (2)	0.10515 (9)	0.0255 (3)	
C5	0.2646 (3)	0.6388 (2)	0.16103 (10)	0.0267 (3)	
H5A	0.1123	0.5896	0.1391	0.032*	
C6	0.3357 (2)	0.6439 (2)	0.24820 (9)	0.0223 (3)	
H6A	0.2326	0.6001	0.2858	0.027*	
C7	0.3278 (4)	0.7036 (3)	0.01100 (10)	0.0412 (4)	
H7A	0.2057	0.6079	−0.0056	0.062*	
H7B	0.4601	0.6835	−0.0227	0.062*	
H7C	0.2628	0.8191	0.0002	0.062*	
C8	−0.0816 (3)	0.82188 (18)	0.77464 (9)	0.0210 (3)	
C9	−0.1916 (2)	0.88215 (18)	0.70096 (9)	0.0211 (3)	
H9A	−0.3353	0.9407	0.7021	0.025*	
C10	−0.0887 (2)	0.85572 (18)	0.62520 (9)	0.0191 (3)	
H10A	−0.1614	0.8961	0.5739	0.023*	
C11	0.1203 (2)	0.77012 (17)	0.62529 (8)	0.0161 (2)	
C12	0.2313 (2)	0.71189 (17)	0.69946 (8)	0.0184 (3)	
H12A	0.3764	0.6553	0.6985	0.022*	
C13	0.1287 (3)	0.73699 (19)	0.77499 (9)	0.0218 (3)	
H13A	0.2014	0.6966	0.8263	0.026*	

### Atomic displacement parameters (Å^2^)

**Table:**

	*U*^11^	*U*^22^	*U*^33^	*U*^12^	*U*^13^	*U*^23^
S1	0.01884 (16)	0.01791 (16)	0.01046 (14)	0.00399 (11)	0.00187 (11)	0.00159 (11)
Cl1	0.0423 (2)	0.0452 (2)	0.02487 (19)	0.01334 (18)	0.01570 (17)	0.00412 (16)
O1	0.0323 (5)	0.0182 (5)	0.0174 (5)	0.0038 (4)	0.0042 (4)	−0.0023 (4)
O2	0.0340 (6)	0.0194 (5)	0.0183 (5)	0.0056 (4)	0.0089 (4)	0.0066 (4)
O3	0.0191 (5)	0.0480 (7)	0.0165 (5)	0.0067 (4)	−0.0019 (4)	0.0021 (4)
N1	0.0186 (5)	0.0198 (5)	0.0155 (5)	0.0024 (4)	0.0016 (4)	0.0022 (4)
C1	0.0190 (6)	0.0143 (5)	0.0120 (5)	0.0029 (4)	0.0017 (4)	0.0012 (4)
C2	0.0200 (6)	0.0294 (7)	0.0162 (6)	−0.0022 (5)	0.0019 (5)	0.0035 (5)
C3	0.0291 (8)	0.0391 (8)	0.0164 (6)	0.0010 (6)	0.0059 (6)	0.0079 (6)
C4	0.0311 (8)	0.0308 (8)	0.0138 (6)	0.0089 (6)	−0.0019 (5)	−0.0004 (5)
C5	0.0219 (7)	0.0341 (8)	0.0215 (7)	−0.0002 (6)	−0.0046 (5)	−0.0021 (6)
C6	0.0194 (6)	0.0282 (7)	0.0193 (6)	−0.0018 (5)	0.0023 (5)	0.0030 (5)
C7	0.0472 (11)	0.0604 (12)	0.0147 (7)	0.0126 (9)	−0.0049 (7)	0.0015 (7)
C8	0.0243 (7)	0.0208 (6)	0.0184 (6)	0.0018 (5)	0.0060 (5)	0.0002 (5)
C9	0.0181 (6)	0.0201 (6)	0.0254 (7)	0.0040 (5)	0.0031 (5)	0.0013 (5)
C10	0.0191 (6)	0.0195 (6)	0.0183 (6)	0.0026 (5)	−0.0017 (5)	0.0032 (5)
C11	0.0179 (6)	0.0147 (6)	0.0155 (6)	0.0005 (4)	0.0016 (5)	0.0010 (4)
C12	0.0185 (6)	0.0182 (6)	0.0185 (6)	0.0041 (5)	−0.0005 (5)	0.0022 (5)
C13	0.0262 (7)	0.0230 (7)	0.0163 (6)	0.0042 (5)	0.0003 (5)	0.0043 (5)

### Geometric parameters (Å, °)

**Table:**

S1—O2	1.4565 (10)	C5—C6	1.387 (2)
S1—O1	1.4572 (10)	C5—H5A	0.9500
S1—O3	1.4587 (11)	C6—H6A	0.9500
S1—C1	1.7682 (13)	C7—H7A	0.9800
Cl1—C8	1.7381 (14)	C7—H7B	0.9800
N1—C11	1.4626 (17)	C7—H7C	0.9800
N1—H1NC	0.920 (12)	C8—C9	1.383 (2)
N1—H1NB	0.916 (12)	C8—C13	1.385 (2)
N1—H1NA	0.925 (12)	C9—C10	1.3894 (19)
C1—C6	1.3880 (19)	C9—H9A	0.9500
C1—C2	1.3892 (18)	C10—C11	1.3817 (18)
C2—C3	1.3875 (19)	C10—H10A	0.9500
C2—H2A	0.9500	C11—C12	1.3850 (18)
C3—C4	1.391 (2)	C12—C13	1.3845 (19)
C3—H3A	0.9500	C12—H12A	0.9500
C4—C5	1.391 (2)	C13—H13A	0.9500
C4—C7	1.510 (2)		
			
O2—S1—O1	111.46 (6)	C5—C6—C1	119.09 (13)
O2—S1—O3	113.20 (7)	C5—C6—H6A	120.5
O1—S1—O3	113.02 (7)	C1—C6—H6A	120.5
O2—S1—C1	106.02 (6)	C4—C7—H7A	109.5
O1—S1—C1	106.15 (6)	C4—C7—H7B	109.5
O3—S1—C1	106.33 (6)	H7A—C7—H7B	109.5
C11—N1—H1NC	110.2 (11)	C4—C7—H7C	109.5
C11—N1—H1NB	111.0 (11)	H7A—C7—H7C	109.5
H1NC—N1—H1NB	108.1 (13)	H7B—C7—H7C	109.5
C11—N1—H1NA	110.6 (11)	C9—C8—C13	121.80 (13)
H1NC—N1—H1NA	108.1 (13)	C9—C8—Cl1	119.03 (11)
H1NB—N1—H1NA	108.8 (13)	C13—C8—Cl1	119.16 (11)
C6—C1—C2	120.72 (12)	C8—C9—C10	118.97 (13)
C6—C1—S1	119.31 (10)	C8—C9—H9A	120.5
C2—C1—S1	119.93 (10)	C10—C9—H9A	120.5
C3—C2—C1	119.31 (13)	C11—C10—C9	119.32 (12)
C3—C2—H2A	120.3	C11—C10—H10A	120.3
C1—C2—H2A	120.3	C9—C10—H10A	120.3
C2—C3—C4	121.00 (14)	C10—C11—C12	121.49 (12)
C2—C3—H3A	119.5	C10—C11—N1	119.76 (12)
C4—C3—H3A	119.5	C12—C11—N1	118.71 (12)
C5—C4—C3	118.61 (13)	C13—C12—C11	119.38 (12)
C5—C4—C7	120.37 (15)	C13—C12—H12A	120.3
C3—C4—C7	121.01 (15)	C11—C12—H12A	120.3
C6—C5—C4	121.26 (14)	C12—C13—C8	119.02 (13)
C6—C5—H5A	119.4	C12—C13—H13A	120.5
C4—C5—H5A	119.4	C8—C13—H13A	120.5
			
O2—S1—C1—C6	32.26 (12)	C4—C5—C6—C1	−0.8 (2)
O1—S1—C1—C6	−86.39 (12)	C2—C1—C6—C5	0.3 (2)
O3—S1—C1—C6	153.01 (11)	S1—C1—C6—C5	177.95 (11)
O2—S1—C1—C2	−150.07 (11)	C13—C8—C9—C10	0.4 (2)
O1—S1—C1—C2	91.28 (12)	Cl1—C8—C9—C10	179.74 (11)
O3—S1—C1—C2	−29.32 (13)	C8—C9—C10—C11	0.0 (2)
C6—C1—C2—C3	0.1 (2)	C9—C10—C11—C12	−0.8 (2)
S1—C1—C2—C3	−177.49 (11)	C9—C10—C11—N1	−178.68 (12)
C1—C2—C3—C4	−0.1 (2)	C10—C11—C12—C13	1.2 (2)
C2—C3—C4—C5	−0.4 (2)	N1—C11—C12—C13	179.09 (12)
C2—C3—C4—C7	178.22 (15)	C11—C12—C13—C8	−0.8 (2)
C3—C4—C5—C6	0.9 (2)	C9—C8—C13—C12	0.0 (2)
C7—C4—C5—C6	−177.77 (15)	Cl1—C8—C13—C12	−179.35 (11)

### Hydrogen-bond geometry (Å, °)

**Table:**

Cg2 is the centroid of the C8–C13 ring.

**Table:**

*D*—H···*A*	*D*—H	H···*A*	*D*···*A*	*D*—H···*A*
N1—H1NC···O1^i^	0.92 (1)	2.02 (1)	2.8579 (16)	151.(2)
N1—H1NC···O1	0.92 (1)	2.42 (2)	3.0814 (16)	129.(1)
N1—H1NB···O3^ii^	0.92 (1)	1.88 (1)	2.7940 (15)	175.(2)
N1—H1NA···O2^iii^	0.93 (1)	1.98 (1)	2.8764 (15)	163.(2)
C2—H2A···Cg2^i^	0.95	2.91	3.5340 (16)	124

Symmetry codes: (i) −*x*+1, −*y*+2, −*z*+1; (ii) *x*−1, *y*, *z*; (iii) −*x*+1, −*y*+1, −*z*+1.
